# Gut microbiome, a novel precision medicine biomarker for hepatocellular carcinoma

**DOI:** 10.3389/fimmu.2025.1568962

**Published:** 2025-12-08

**Authors:** Pin-Jung Chen, Suzanne Devkota, Stephen Shiao, Andrew Hendifar, Ju Dong Yang

**Affiliations:** 1 Department of Hematology/Oncology, Cedars-Sinai Medical Center, Los Angeles, CA, United States; 2 Human Microbiome Research Institute, Cedars-Sinai Medical Center, Los Angeles, CA, United States; 3 Karsh Division of Gastroenterology and Hepatology, Cedars-Sinai Medical Center, Los Angeles, CA, United States; 4 Department of Radiation Oncology, Cedars-Sinai Medical Center, Los Angeles, CA, United States

**Keywords:** gut micobiome, hepatocellualr carcinoma, immunotherapy, gut-liver axis, presicion oncology

## Abstract

Hepatocellular carcinoma (HCC) remains a leading cause of cancer-related mortality worldwide. Although immune checkpoint inhibitors (ICIs) have transformed systemic therapy, durable responses are achieved in only a subset of patients, highlighting the need for reliable predictive biomarkers. The gut–liver axis, a bidirectional network linking intestinal microbiota, microbial metabolites, and hepatic immune pathways, has emerged as a key regulator of liver immunity and tumor progression. Growing evidence indicates that the gut microbiome modulates ICI efficacy by shaping immune activation, cytokine signaling, and drug metabolism. This review summarizes current insights into how gut microbial composition and metabolites influence immunotherapy outcomes in HCC and discusses microbiome-targeted strategies, including fecal microbiota transplantation (FMT), prebiotics, probiotics, and dietary interventions. Further research and clinical validation are needed before these insights can be effectively integrated into HCC management.

## 1 Introduction

Liver cancer is the third leading cause of cancer-related mortality worldwide, accounting for over 860,000 new cases and approximately 750,000 deaths per year (1). Hepatocellular carcinoma (HCC) represents the most common primary liver cancer and a substantial global health burden (2).

Programmed cell death protein 1 (PD-1) is an immune checkpoint receptor expressed on T cells that plays a crucial role in regulating immune responses. By binding to its ligands PD-L1 or PD-L2, often upregulated on tumor cells, PD-1 inhibits T cell activation, allowing cancer cells to evade immune surveillance and promoting tumor progression. Inhibiting the PD-1 pathway with immune checkpoint inhibitors (ICIs) restores anti-tumor immunity and has become a cornerstone of modern cancer immunotherapy (3–7). ICI-based immunotherapy has also revolutionized the treatment of HCC (8). Atezolizumab plus bevacizumab and tremelimumab plus durvalumab are approved as standard-of-care for advanced HCC patients in the first line setting (9, 10). While these therapies can lead to remarkable tumor regression in a subset of patients, some patients experience little to no benefit. Furthermore, most patients with advanced HCC do not derive durable response to ICIs, highlighting the critical need for the development of predictive biomarkers that can guide personalized immunotherapy strategies.

The liver, intricately connected to the gut microbiome and bacterial metabolites via the portal vein, hosts a dense network of immune cells. This gut–liver axis represents a bidirectional communication system through which gut-derived microbial products and metabolites reach the liver via the portal circulation, while bile acids, cytokines, and immune mediators are secreted back into the gut. This close interplay regulates hepatic immune tolerance, metabolic homeostasis, and inflammation, placing the gut microbiome as an important potential regulator of hepatic inflammation, the tumor microenvironment, and anti-tumor immune responses (11–14).

In 2015, preclinical mouse studies (MCA205 sarcoma, Rat melanoma, and MC38 colon cancer) showed that gut microbiota composition could influence the success of immunotherapy, as well as spontaneous anti-tumor immunity. In these studies, the efficacy of ICIs varied with the microbial makeup in the mice, underscoring the potential of the gut microbiome as a predictive biomarker of cancer treatment responses (15, 16). Further studies have shown that antibiotics, by altering the composition and diversity of the gut microbiome, can disrupt this balance and impact immune response. Both animal and human studies suggest that gut microbiome changes correlate with the effectiveness of immunotherapy in cancers such as melanoma, non-small cell lung cancer, colorectal cancer, and renal cell carcinoma (17–23). Interestingly, the microbial profiles observed in responders were also associated with frequent immune-related colitis (24).

**Figure 1:**
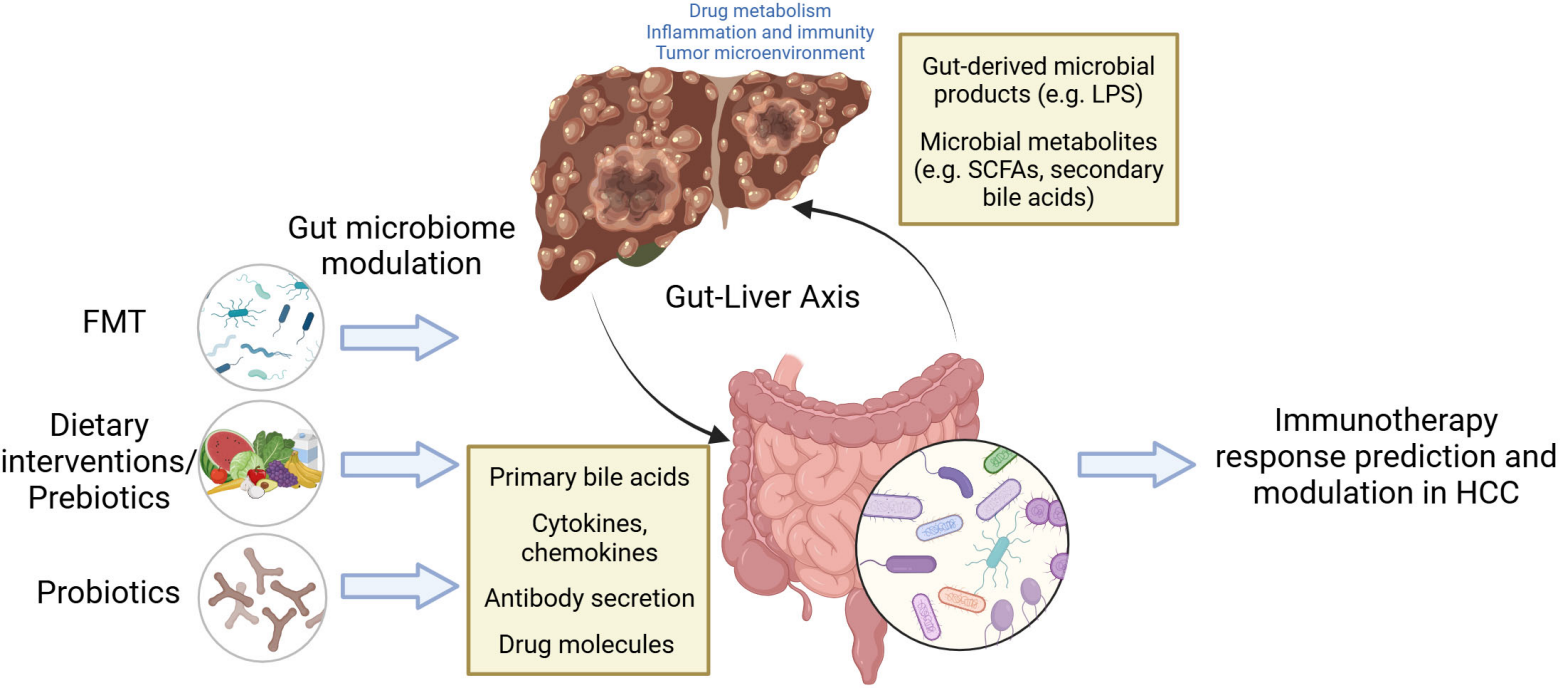
The role of gut microbiome in immunotherapy response prediction and modulation for HCC. The illustration highlights the gut-liver anatomical connection and bidirectional interactions. While our review focuses primarily on the influence of the gut on hepatic immunity, tumor microenvironment, and cancer response, reciprocal signaling pathways from the liver to the gut (e.g., via primary bile acids) are acknowledged but not detailed in the text of this review.

Recently, emerging data have also linked the gut microbiome to treatment responses prediction and prognosis in HCC. In this review, we summarize the current literature on the role of the gut microbiome in modulating immunotherapy outcomes in HCC and explore its potential as a precision medicine biomarker for more effective, personalized cancer treatment approaches (**Figure 1**).

## 2 Impact of gut microbiome on immunotherapy response

The gut microbiome plays a crucial role in modulating the host immune system, which in turn affects the response to immunotherapy (25). Early evidence showed that the use of antibiotics before or shortly after initiating ICIs led to reduced treatment efficacy in advanced-stage lung, kidney, and bladder cancers, although the impact of antibiotic exposure on treatment outcomes in HCC patients remains unclear. Routy et al. hypothesized that this relationship reflects gut dysbiosis, an imbalance or disruption in the normal composition and function of the gut microbiota, resulting from transient antibiotic-induced changes in microbial composition and diversity (18). In addition, patients with primary resistance to ICIs had an imbalance in gut microbiota composition, while responders exhibited enrichment of particular bacterial species, notably *Akkermansia muciniphila* (18, 26–29). In this study, fecal microbiota transplantation (FMT) from ICI responders into germ-free mice restored treatment efficacy to PD-1 blockade, while FMT from non-responders did not. *Akkermansia muciniphila* was identified as a key bacterium associated with positive outcomes. Mechanistically, oral supplementation with *Akkermansia muciniphila* promoted immune cell recruitment, such as CXCR3^+^CD4^+^ T lymphocytes and CD8^+^ T lymphocytes, enhancing antitumor immunity. This effect was mediated through interleukin-12 (IL-12), a key cytokine that activates dendritic cells, stimulates T cells, and increases interferon-gamma (IFN-γ) production. These findings suggest that gut microbiota composition not only correlates with, but can actively modulate, responses to cancer immunotherapy by influencing immune cell recruitment and activation, and could therefore be targeted to improve treatment efficacy (18, 30).

Building on these findings, several studies in HCC have demonstrated that gut microbiome composition and metabolites are associated with outcomes of ICI-treated unresectable HCC (26–29, 31). Using stool metagenomic sequencing, Chung et al. found that the gut microbial compositions could serve as a predictor of treatment outcomes of nivolumab in HCC patients. Specific bacterial taxa such as *Akkermansia* sp*ecies* were enriched in responders, while non-responders had a skewed *Firmicutes/Bacteroidetes* ratio (< 0.5 or > 1.5) and lower *Prevotella/Bacteroides* ratios (28). Similarly, Ponziani et al. evaluated 11 patients treated with a combination of tremelimumab and durvalumab, or with monotherapy using either agent. Consistent with Chung et al, this study also found enrichment of *Akkermansia muciniphila* and reduced *Enterobacteriaceae*, along with lower levels of fecal calprotectin, were associated with better treatment outcomes. Together, these results support the idea that gut microbiota profiling, combined with inflammatory markers, may serve as a potential biomarker for predicting the response to ICI-based immunotherapy in HCC patients (29).

To explore microbial and metabolomic interactions further, Wu et al. investigated the fecal and serum samples from 35 patients with unresectable HCC who received anti-PD1-based immunotherapy. Using 16S rRNA sequencing, the microbiome profile was analyzed at baseline and prior to each anti–PD-1 infusion, enabling longitudinal assessment of microbiome dynamics in relation to treatment response. Responders exhibited increased abundance of *Faecalibacterium* and *Ruminococcus*, while non-responders had an increase in potentially pathogenic bacteria like *Klebsiella* (31). The findings were similar to those observed by Zheng et al. based on metagenomic sequencing of fecal samples from eight HCC patients collected at baseline, one week after treatment initiation, and on the day of each treatment (27). Metabolomic profiling revealed that, besides the specific bacterial genera, certain metabolites, such as alpha-D-glucose, were also linked to treatment response. The classifier integrating gut microbial and serum metabolite data can help discriminate HCC patients who might benefit from immunotherapy (31), suggesting a functional link between microbial composition, metabolite production, and immune modulation.

Across multiple studies, a common finding is that fecal samples from HCC patients responding to ICI exhibited higher gut microbial diversity and stability than those of non-responders (27, 28, 31). In contrast, non-responders experienced a shift in microbial composition. Zheng et al. reported that a marked increase in *Proteobacteria*, specifically *Escherichia coli*, as early as week 3 and became predominant by week 12, was associated with poorer outcomes, whereas responders maintained stable levels of beneficial bacteria such as *Akkermansia muciniphila* and *Ruminococcaceae* sp*ecies*. These dynamic variations suggest that early changes in gut microbiota have distinct implications for drug efficacy and disease prognosis (27).

Most human studies are still underpowered for microbiome analyses at this stage. Although there is limited reproducibility across different cohorts or studies, multiple datasets suggests that *Akkermansia muciniphila* positively influences immunotherapy responses by enhancing antitumor immunity through the recruitment of CD8^+^ T cells and reduction of immunosuppressive regulatory T cells (Tregs), thereby creating a more favorable tumor immune microenvironment (18, 26, 27, 31–33). *Akkermansia muciniphila* is a Gram-negative, anaerobic bacterium that resides in the mucus layer of the human gut and helps degrading mucin. It has attracted a growing interest due to its beneficial effects on health (34–36). Its enrichment is associated with greater gut microbial diversity, which supports a stable and effective immune response during PD-1 blockade (27). Additionally, *Akkermansia muciniphila* improves gut barrier integrity, reduces inflammation, and promotes the production of metabolites such as short-chain fatty acids (SCFAs), including butyrate, acetate, and propionate that further modulate T cell activation and differentiation. SCFAs have well-characterized, multifaceted roles in shaping both immune responses and hepatic environment: butyrate, a 4-carbon SCFA, enhances the differentiation of peripheral Tregs by increasing Foxp3 expression via histone acetylation and epigenetic regulation (37, 38), while also boosting cytotoxic CD8^+^ T cell function by enhancing T cell receptor signaling and upregulating granzyme B and IFN-γ production (32, 33). Moreover, SCFAs help maintain intestinal homeostasis and barrier integrity and modulate cytokine production, contributing to systemic immune regulation and improved responses to ICIs in HCC (39, 40).

## 3 Influence of gut microbiome on drug metabolism

The human gut microbiome contains 10–100 trillion symbiotic bacteria that play a crucial role in altering host metabolism by utilizing drugs as substrates in enzymatic processes (41). Gut microbiota affects the drug metabolism in HCC both directly and indirectly. The complex ecosystem of microorganisms can alter the bioavailability, efficacy, and toxicity of drugs, influencing treatment outcomes (42).

The gut microbiota can directly metabolize certain drugs through various enzymatic reactions such as reduction, acetylation/deacylation, decarboxylation, dehydroxylation, demethylation, dehalogenation, and conjugates hydrolysis (43). For example, the gut microbiota hydrolyzes conjugated irinotecan metabolite SN-38 glucuronide, leading to the release of active SN-38, which can cause severe intestinal toxicity (44–46). More importantly, the microbiome indirectly affects drug metabolism by modulating the expression and activity of host drug-metabolizing enzymes and transporters in the liver and intestine, particularly cytochrome P450 enzymes (47). This modulation can significantly impact the pharmacokinetics of drugs used in HCC treatment. Clinical observations have linked antibiotics use and gut microbiome disruption to worse outcomes in HCC patients treated with drugs like sorafenib (48). The impact of antibiotics on immunotherapy efficacy in HCC is still being debated as some studies have shown conflicting results (49–52). While certain gut microbial metabolites promote immune activation and drug metabolism, dysbiosis can have the opposite effect by disrupting bile-acid signaling, hepatic cytochrome P450 activity, and drug transport, resulting in variable drug exposure and diminished treatment efficacy (53, 54).

A significant aspect of the microbiome’s influence on HCC and immunotherapy is its role in gut microbiota-derived metabolite metabolism. The gut microbiome produces metabolites that can compete with drugs for metabolism pathways, altering the bioavailability, efficacy, and toxicity of immunotherapeutic agents. Bile acids, primarily produced in the liver, affect the enterohepatic recycling of drugs, influencing their efficacy and side effects. The microbiome can mediate the metabolism of primary into secondary bile acids, which are recycled back to the liver through enterohepatic circulation. This feedback loop between the gut and liver through the microbiota-bile acid interaction is essential for maintaining metabolic balance (11, 47). SCFAs, produced by bacterial fermentation of dietary fiber, have been shown to regulate cytochrome P450 enzymes (55), which impact the metabolism of drugs used in HCC treatment. For example, butyrate, enhances gut motility and limits the growth of pathogenic microorganisms. SCFAs are most abundant in the proximal colon. As the primary energy source of colonic epithelial cells, butyrate can affect the expression and function of drug metabolizing enzymes, such as CYP1A1 and CYP1B1, in colonic epithelial cells and mediate drug metabolism in the gut. Most commensal bacteria known to produce relatively high levels of butyrate in the gastrointestinal tract belong to the *Clostridium* cluster of the phylum *Firmicutes*, such as *Faecalibacterium prausnitzii*, *Eubacterium* sp*ecies*, *Roseburia* species, *Anaerostipes* sp*ecies, Ruminococcus* sp*ecies, Coprococcus* sp*ecies, Subdoligranulum variabile* (56–60). SCFAs can also affect phase II drug-metabolizing enzymes, such as UDP-glucuronosyltransferases and glutathione S-transferases. Using 16S rRNA sequencing and metagenomics shotgun sequencing, Peng et al. identified specific SCFA-producing bacterial taxa, such as *Eubacterium, Lactobacillus, Streptococcus, Ruminococcaceae, Lachnospiraceae*, and *Prevotellaceae*, as positively associated with anti-PD-1/PD-L1 treatment response in 74 patients with advanced gastrointestinal cancers. The taxonomic features were used to build machine learning classifiers and evaluated for predictive utility to stratify patients treated with immunotherapy through area under curve (AUC) analysis from receiver operating characteristic (ROC) curves. The predictive performance of the classifier was also independently validated in two published melanoma cohorts, demonstrating consistent stratification of responders (61).

## 4 Modulation of inflammation and immunity by gut microbiome

The gut microbiome shapes the development and regulation of innate and adaptive immunity, which is central to the efficacy of immunotherapy in HCC. The composition of the commensal microbiota affects the presence and activity of cytotoxic T cells, natural killer (NK) cells, Tregs, myeloid-derived suppressor cells (MDSCs), and tumor associated macrophages (TAMs) (11, 62). The interactions between the gut microbiome, its metabolites, and the host immune system significantly influence inflammation and immunity in the liver, ultimately affecting carcinogenesis, HCC progression, and treatment outcomes (12).

The composition and function of the gut microbiome can significantly impact intestinal barrier integrity. The bacterial metabolites, such as SCFAs and bile acids, can influence intestinal barrier function and regulate hepatic inflammation and immunity. Dysbiosis can lead to increased intestinal permeability and translocation of bacterial products, such as lipopolysaccharides (LPS), into the bloodstream (14). The presence of these bacterial components in circulation can promote chronic low-grade inflammation, that may interfere with treatment efficacy (63, 64).

Gut microbiota-derived metabolites are key mediators in modulating immunotherapy outcomes in HCC. They can both enhance T cell-mediated antitumor responses and affect the occurrence and severity of immune-related adverse events (irAEs). The SCFAs, particularly butyrate, acetate, and propionate, have significant immunomodulatory effects (39). Specific members of the microbial community have been found to potentiate the generation of anti-inflammatory Tregs or pro-inflammatory T helper _17_ (TH_17_) cells. Butyrate, for instance, has been shown to enhance the differentiation of Tregs, which maintains immune tolerance and preventing autoimmune responses (37, 38), and the activity of cytotoxic CD8^+^ T cells, which upregulates the anticancer immunity (32, 33). Additionally, SCFAs can influence the overall inflammatory state of the gut by modulating the production of cytokines and enhancing the integrity of the intestinal barrier (40).

The impact of bacterial metabolites extends beyond the gut and can influence systemic immune responses. The gut microbiota metabolites have been found to enhance T cell-mediated antitumor responses by recruiting CXCR3^+^ CD4^+^ T lymphocytes to the tumor site, triggering cytokine production, and promoting dendritic cell activity, which are particularly relevant in the context of cancer immunotherapy (18). These metabolites can affect T cell differentiation, activation, and effector functions, potentially improving the efficacy of ICIs.

Bile acids, another important class of metabolites influenced by the gut microbiome, play a significant role in regulating inflammation and immunity. Primary bile acids synthesized in the liver are metabolized by gut bacteria into secondary bile acids which serves as signaling molecules that activate nuclear receptors such as the Farnesoid X Receptor (FXR) and G protein-coupled bile acid receptor 1 (TGR 5) (65, 66). FXR regulates bile acid synthesis and metabolism, activation and has been shown to have anti-inflammatory effects in the liver (67). Researchers found that patients with HCC and HCC mouse models had reduced levels of secondary bile acids, particularly conjugated deoxycholic acid (DCA), and lower levels of bile salt hydrolase (BSH)-producing gut bacteria. Experiments with vancomycin-treated mice further confirmed that reduced BSH-producing bacteria and secondary bile acids promote liver tumor growth (66).

In addition to SCFAs and bile acids, other gut microbial metabolites, including tryptophan derivatives, polyamines, and trimethylamine N-oxide (TMAO), also modulate inflammation and immunity in HCC. Tryptophan-derived indoles can activate the aryl hydrocarbon receptor (AhR), promoting mucosal immune tolerance and shaping hepatic immune homeostasis. While AhR activation maintains mucosal immune balance under physiological conditions, its sustained activation in HCC fosters immune suppression and tumor progression (68–70). Polyamines produced by gut microbes promote macrophage M2 polarization and an immunosuppressive tumor microenvironment (71). Elevated TMAO levels, derived from microbial choline metabolism, have been shown to promote inflammation and tumor growth in HCC (72). Collectively, these findings highlight that a wide array of microbial metabolites affect host immunity and may represent novel therapeutic targets for modulating HCC immunotherapy responses.

In conclusion, the gut microbiome’s influence on inflammation and immunity is multifaceted. Through the production of metabolites like SCFAs and secondary bile acids, modulation of intestinal barrier function, and direct interactions with immune cells, the microbiome shapes both local and systemic immune responses. The gut-liver axis, mediated by bacterial metabolites and immune signaling, plays a crucial role in liver disease progression and response to therapy.

## 5 Effect of gut microbiome on tumor microenvironment

The tumor microenvironment (TME) is a complex and dynamic ecosystem surrounding tumors, comprising various components that interact with cancer cells to influence tumor growth, progression, and treatment response. These components include immune cells, the extracellular matrix, blood vessels, and stromal cells such as cancer-associated fibroblasts. The TME plays a crucial role in tumor development through bidirectional interactions: tumors shape their microenvironment to support growth and immune evasion, while the microenvironment influences tumor behavior, metastasis potential, and treatment resistance. Key processes within the TME include immune modulation, ECM remodeling, angiogenesis, and stromal cell recruitment (62). Understanding and targeting these complex interactions within the TME has become a significant focus in cancer research and therapy development, as disrupting these supportive elements can potentially weaken tumors and enhance the effectiveness of treatments like immunotherapy (73, 74).

The gut microbiome may regulate the TME of HCC through several mechanisms, including modulating the composition and function of immune cells and the production of cytokines and chemokines (11, 75). Commensal genera such as *Akkermansia*, *Bifidobacterium*, *Faecalibacterium*, and *Ruminococcus*, along with the production of metabolites such as SCFAs can enhance T cell-mediated antitumor responses, while pro-inflammatory taxa including *Escherichia/Shigella*, *Enterococcus*, and *Klebsiella* may promote immunosuppressive cells like Tregs and MDSCs to help tumor immune evasion (12, 38, 62). This balance between pro-inflammatory and anti-inflammatory processes is critical within TME (73). Beyond immune modulation, the microbiome also affects angiogenesis and ECM remodeling (76–79). Moreover, microbial metabolites, including SCFAs and secondary bile acids, can directly impact cancer cell proliferation, survival, and metastatic potential (80–82).

HCC is often linked to cirrhosis and arises within a microenvironment marked by pronounced inflammation, immune system changes, and significant cellular abnormalities. Huang et al. demonstrated that alterations in the tumor immune microenvironment, driven by gut microbiota through serum bile acids, may be associated with tumor burden and unfavorable prognosis in HCC (83).

A study by Ma et al. revealed that bile acid metabolism can affect the liver’s immune microenvironment by either promoting or inhibiting the accumulation of CXCR6^+^ NK T cells. Primary bile acids could foster NK T cell activation and anti-tumor immunity by promoting the expression of the chemokine CXCL16, whereas secondary bile acids had opposing effects. In mouse models, altering the gut microbiome through antibiotics or specific bile acid supplementation effectively influenced liver cancer progression, highlighting a potential therapeutic pathway for liver cancer through microbiome modulation (11).

Understanding these complex interactions has led to the exploration of microbiome-based therapies, including fecal microbiota transplantation and the use of specific bacterial strains or their products to enhance immunotherapy efficacy (11, 12). Future research in this area may lead to the development of novel strategies to improve HCC treatment outcomes by targeting the gut-liver axis.

## 6 Biomarker potential of gut microbiome

The composition and diversity of gut microbiome is becoming increasingly recognized for its role in the regulation of immunity and as potential biomarkers for predicting treatment response in HCC (63). Emerging studies in hepatobiliary cancers have demonstrated the correlation between certain microbial profiles and improved treatment efficacy, progression-free survival (PFS), and overall survival (OS), while other taxa are associated with poorer outcomes (26–29, 31, 84, 85) (**Table 1**). Additionally, gut microbial signatures and diversity impact the severity irAEs, such as colitis (85). These could potentially help identify patients most likely to derive the greatest benefit from immunotherapy (42, 86).

**Table 1:** Microbial signatures associated with immunotherapy treatment response in HCC.

Microbiome predictors enriched	ICIs/patients included/number of patients	Method used	Reference
R: *Lachnoclostridium, Lachnospiraceae, Veillonella*, ursodeoxycholic acid, and ursocholic acid NR: *Prevotella 9*	Nivolumab and Pembrolizumab/HCC/74	16S rRNA sequencing	Lee et al. (26)
R: *Akkermansia muciniphila, Ruminococcaceae* sp.*, and probiotic lactic acid bacteria (e.g. Lactobacillus* sp.*, Bifidobacterium dentium, Streptococcus thermophilus)* NR: Increase of *Proteobacteria* (*Escherichia coli*) in dynamic analysis	Camrelizumab/HCC/8	Metagenomic sequencing	Zheng et al. (27)
R: *Akkermansia* sp.*, Citrobacter freundii, Azospirillum* sp.*, and Enterococcus durans*, NR: *Proteobacteria* (e.g. *Escherichia coli*), skewed *Firmicutes/Bacteroidetes* Ratio (<0.5 or >1.5), lower *Prevotella/Bacteroides* ratio	Nivolumab/HCC/8	16S rRNA sequencing	Chung et al. (28)
R: *Akkermansia muciniphila, Bifidobacterium* NR: *Enterobacteriaceae*, fecal calprotectin	Tremelimumab and/or Durvalumab/HCC/11	16S rRNA sequencing	Ponziani et al. (29)
R: *Faecalibacterium, Blautia, Lachnospiracea incertae Sedis, Megamonas, Ruminococcus, Coprococcus, Dorea, Haemophilus*, and Alpha-d-Glucose (at 3 months of treatment) NR: *Atopobium, Leptotrichia, Campylobacter, Allisonella, Methanobrevibacter*, *Parabacteroides, Bifidobacterium, and Lactobacillus*	Anti-PD-1 based systemic therapy/HCC/35	16S rRNA sequencing	Wu et al. (31)
R: *Collinsella genus, Ruminococcus* sp. NR: *Bacteroides, Veillonella* sp.	Anti-PD-1-based combination therapy/HCC/45	Whole genome shotgun strategy	Xin et al. (84)
R: *Lachnospiraceae bacterium-GAM79, Alistipes* sp. *Marseille-P5997, Ruminococcus calidus, Faecalibacterium genus, Erysipeotrichaceae bacterium GAM147* NR: *Veillonellaceae*	Anti-PD-1 based systemic therapy/HCC and biliary tract cancer/65	Metagenomic sequencing	Mao et al. (85)

R, responder group; NR, non-responder group; ICI, immune checkpoint inhibitor.

The mechanisms by which the gut microbiome influences immunotherapy responses are multifaceted, involving modulation of metabolic pathways, regulation of immune activity, and influence on inflammation levels. These interactions underscore the complex interplay between the microbiome and treatment outcomes. Recent research has focused on combining microbial signatures with metabolomic data to enhance the predictive power of gut microbiome-based biomarkers. Wu et al. used machine learning models based on gut microbial and serum metabolomic data to predict immunotherapy outcomes, with the serum metabolite-based classifier showing higher predictive accuracy than gut microbiota alone (31).

Microbe-associated transcripts in host tumors might also provide partial insights into how gut microbiota contributes to HCC pathogenesis. Huang et al. investigated the interplay between gut microbiota, liver transcriptome, and clinical outcomes in patients with HBV-related HCC. Integrative analysis identified significant associations between these microbes and host gene expression profiles, implicating the tumor immune microenvironment and bile acid metabolism as mediators of gut-liver communication. Certain microbial markers were predictive of clinical prognosis, achieving an AUC of 81% for survival prediction using machine learning models (83).

Despite the promising potential of the gut microbiome as a biomarker for immunotherapy response in HCC, several challenges remain. The dynamic nature of the microbiome, which can change during treatment, necessitates longitudinal monitoring. Further research is needed to validate the predictive power of microbial signatures across larger and more diverse patient populations. Additionally, combining gut microbial signatures with metabolomic data, clinical factors, and other biomarkers, such as tumor genomic features and PD-L1 expression, may provide more comprehensive predictive models in HCC immunotherapy. Developing standardized protocols for sample collection, processing, and analysis is crucial for the clinical application of gut microbiome-based biomarkers.

In conclusion, the gut microbiome shows great promise as a potential biomarker for predicting immunotherapy response in HCC patients. As our understanding of the gut microbiome’s role in cancer treatment continues to evolve, it holds the potential to significantly improve patient outcomes and advance the field of precision oncology.

## 7 Microbiome-targeted strategies to modulate the treatment efficacy

Increasing evidence has demonstrated microbiome involvement in the beneficial regulation of the efficacy of therapeutics that stimulate anticancer responses. As research in this field progresses, understanding the intricate interplay between gut microbiota and drugs open up possibilities for developing microbiome-based therapies as adjuvants to enhance HCC treatment outcomes. Researchers are exploring various approaches to modulate the gut microbiome through dietary interventions, prebiotics, probiotics, and FMT to aid in the treatment of disease (25, 63). However, these approaches are likely to require adaptation depending on the cancer type and therapeutic drug type. While evidence from other cancer types highlights the potential of harnessing these interventions, further research is needed to establish their applicability and effectiveness in HCC (**Table 2**). This could represent a paradigm shift in the management of HCC, improving outcomes for patients undergoing immunotherapy.

**Table 2:** Microbiome-targeted strategies to modulate the treatment efficacy.

Intervention	Impact on gut microbiome	Key preclinical/clinical findings
Fecal Microbiota Transplantation (FMT)	- Introduces diverse and balanced microbial communities from treatment responders to overcome treatment resistance. - Reduces *Proteobacteria*-linked dysbiosis and improves immune cell recruitment.	- While studies specifically for FMT in HCC immunotherapy are limited, ongoing clinical trials (FAB-HCC, NCT05750030 and FLORA, NCT05690048) aim to explore the potential of FMT in enhancing the treatment efficacy of HCC immunotherapy. - Shows promise as a steroid-sparing option for managing immunotherapy-induced colitis.
Dietary Intervention	- Diets that modify gut microbiota composition and promote SCFA production to enhance the efficacy of immunotherapy. - Bioactive metabolites influence inflammation and antitumor immunity.	- High-fiber, Mediterranean, and ketogenic diets are linked to improved immune responses in various types of cancers. - Personalized approaches are necessary. Future research should focus on identifying specific dietary components that modulate the gut microbiome to enhance immunotherapy efficacy in HCC.
Prebiotics	- Non-digestible food ingredients that selectively promote the growth of beneficial microbes such as SCFA producers while reducing harmful strains to enhance gut barrier integrity and support antitumor immune responses.	- Enhances the efficacy of cancer treatments by fostering a favorable gut environment in preclinical models. - Personalized approaches are necessary. Future research should focus on identifying specific prebiotics that modulate the gut microbiome to enhance immunotherapy efficacy in HCC.
Probiotics	- Introduces beneficial live bacteria, e.g., *Akkermansia muciniphila* and *Roseburia intestinalis*, to restore gut barrier function and boost immune responses.	- *Akkermansia muciniphila* supplementation was shown to restore anti-PD-1 responses in preclinical models. - Probiotic mixtures like Prohep were shown to suppress HCC growth in preclinical models. - An ongoing clinical trial, NCT05032014, is investigating the potential of probiotics to enhance the efficacy of PD-1 inhibitors in liver cancer patients.

### 7.1 Fecal microbiota transplantation

In the United States., the Food and Drug Administration (FDA) only approved FMT for recurrent *Clostridioides difficile* infection (CDI), so most studies using FMT to enhance cancer immunotherapy efficacy are exploring under Investigational New Drug (IND) (87, 88).

FMT introduces a diverse and balanced microbial community from other individuals, usually from ICI responders, via an oral lyophilized pill, upper GI endoscopy, colonoscopy, or enema. Studies have demonstrated that FMT from immunotherapy responders to germ-free or antibiotic-treated mice can enhance tumor control and improve antitumor immune responses to ICIs (89). This approach has shown promise in clinical settings as well. In 2021, two clinical trials in melanoma patients demonstrated that FMT from ICI responders, combined with anti-PD-1 therapy, could overcome resistance to PD-1 blockade (90, 91). FMT also shows promise as a treatment for colitis in patients undergoing immunotherapy. Wang et al. evaluated FMT as a first-line therapy for immune-mediated colitis caused by immunotherapy, 71.4% of the seven patients experienced symptom relief within a day, and 85.7% were able to resume cancer treatment. These findings suggest that FMT could be a safe and effective steroid-sparing option for managing immunotherapy-induced colitis (92). Additionally, FMT has been reported effective as a salvage therapy for refractory ICI-related colitis in case series. Dysbiotic shift significant for *Proteobacteria* expansion was observed to be characteristic of ICI-related colitis at the time of symptom onset and could be salvaged by FMT (93). Its benefits may stem from restoring the gut microbiome and increasing the proportion of regulatory T cell levels in the gut mucosa, potentially mitigating ICI-related colitis (94).

While studies specifically for FMT in HCC immunotherapy are limited, ongoing clinical trials hope to provide a foundation for exploring FMT as a potential strategy in HCC immunotherapy. For example, an ongoing phase IIa clinical trial (NCT05750030), FAB-HCC pilot study, is evaluating the combination of FMT with Atezolizumab and Bevacizumab in patients with advanced HCC who failed to respond to prior immunotherapy. Conducted in Vienna, Austria, the trial aims to assess the safety and efficacy of this combination. The primary objective is to assess treatment-related adverse events, while secondary objectives include best radiological response, objective response rate (ORR), disease control rate (DCR), PFS, OS, and quality of life measures. Additionally, exploratory objectives involve analyzing gut microbiota changes and immune activity post-FMT. The trial plans to enroll 12 patients and has a planned duration of 48 months.

The FLORA (Fecal Microbiota Transfer in Liver Cancer to Overcome Resistance to Atezolizumab/Bevacizumab) trial, registered as NCT05690048, is another pioneering phase II clinical study that aims to explore the potential of FMT in enhancing treatment efficacy for HCC patients who receive Atezolizumab and Bevacizumab. The study’s premise is rooted in the growing body of evidence suggesting a crucial role of the gut microbiome in modulating responses to cancer immunotherapies, particularly ICIs. Based in Germany, the primary outcomes include differential tumoral CD8 T-cell infiltration and adverse events. The secondary outcomes include PFS, OS, and change in hepatic function.

While specific results from FAB-HCC pilot study and FLORA are not yet available, these efforts represent a significant step forward in personalized cancer treatment, potentially offering a new strategy to enhance the effectiveness of immunotherapy and to overcome immunotherapy resistance in liver cancer patients and paving the way for microbiome-based interventions.

### 7.2 Dietary interventions and prebiotics

Dietary modifications and prebiotic supplementation represent another avenue for modulating the gut microbiome to enhance immunotherapy efficacy. While specific studies on HCC are limited, research in other cancer types has shown that dietary interventions can alter the gut microbiome composition and promote the production of SCFAs, particularly butyrate, and potentially influence immunotherapy outcomes (95–98). Prebiotics, which are non-digestible food ingredients that selectively promote the growth of beneficial bacteria, may also play a role in enhancing the efficacy of cancer immunotherapies.

Diet is one of the most significant modifiable factors affecting gut microbiota. Various dietary patterns, such as high-fiber diets, Mediterranean diets, and ketogenic diets, have been shown to promote the growth of beneficial bacteria while inhibiting pathogenic strains and be metabolized by intestinal bacteria into bioactive metabolites depending on the nutrient composition, which are linked to improved immune responses in various types of cancers (97, 99–102). In the context of HCC, mounting evidence has also shown that dietary patterns are associated with risk and prognosis of HCC, potentially due to alterations in microbial composition and diversity, thereby affecting the production of metabolites such as SCFAs that support antitumor immunity and gut barrier integrity (103–109). Changes in microbiota driven by diet can impact bile acid metabolism and promote hepatic inflammation through increased translocation of microbial products. These effects could collectively influence immune cell activation within the liver, shaping the tumor microenvironment and affecting tumor development and progression (110).

Prebiotics serve as substrates for beneficial bacteria. The administration of prebiotics is intended to selectively support the growth of beneficial microorganisms, promoting the production of SCFAs and enhancing gut barrier integrity, compared to the direct application of microbiotics. Commonly studied prebiotics include inulin, fructooligosaccharides (FOS), galactooligosaccharides (GOS), and resistant starch, all of which enrich SCFA-producing taxa such as *Bifidobacterium*, *Lactobacillus*, and *Faecalibacterium*. Studies have shown that prebiotic supplementation can increase butyrate levels, enhance dendritic-cell maturation, and improve CD8^+^ T-cell activation, thereby augmenting the efficacy of cancer treatments by in preclinical models (111, 112).

Because the gut microbiome varies significantly among individuals, influenced by diet, environmental factors, and genetics, personalized approaches are necessary. Future research should focus on identifying specific dietary components and prebiotics that modulate the gut microbiome to enhance immunotherapy efficacy in HCC. Integrating microbiome profiling into clinical practice could enable the development of personalized strategies to maximize the benefits of these interventions. Additionally, the long-term safety and feasibility of these interventions must be established, particularly in patients with compromised liver function.

### 7.3 Probiotics

Probiotics are defined as live microorganisms that confer health benefits when administered in adequate amounts. The administration of specific bacterial strains or probiotics has shown the potential in enhancing immunotherapy efficacy (113). A study explored the role of *Roseburia intestinalis*, a butyrate-producing bacterium, in improving colorectal cancer (CRC) outcomes and enhancing anti-PD-1 immunotherapy efficacy. The researchers found that *Roseburia intestinalis* is significantly depleted in CRC patients and demonstrated that its administration inhibits tumor growth in mouse models by restoring gut barrier function and boosting immune responses. *Roseburia intestinalis* and its metabolite butyrate hold potential as adjunct therapies to improve CRC treatment outcomes, particularly for patients resistant to anti-PD-1 immunotherapy (114). While the study focused on CRC, it highlighted the potential for combining probiotics with cancer treatments, which could also be explored in the context of HCC immunotherapy.

In HCC, certain bacterial species have been identified as potentially beneficial. For instance, *Akkermansia muciniphila* is associated with improved responses to anti-PD-1 therapy as discussed earlier in this review (18, 27–29). Supplementation with *Akkermansia muciniphila*, either alone or in combination with other bacteria like *Enterococcus hirae*, has been shown to restore responses to PD-1 blockade in preclinical models. Probiotics can bind carcinogens, enhance gut barrier integrity, reduce bacterial translocation, and modulate the production of anti-inflammatory metabolites like SCFAs (101, 115, 116). Experimental evidence suggests probiotics’ ability to inhibit HCC progression in animal models by reducing tumor growth, suppressing inflammatory cytokines, and promoting a more balanced microbiome composition. Li et al. reported that feeding a novel probiotic mixture, Prohep (comprising *Lactobacillus rhamnosus GG*, *Escherichia coli Nissle 1917* and heat-inactivated VSL#3), to tumor-injected mice could shift the gut microbiota composition and reduce the size of liver tumors. In addition to the reduction of tumor size, angiogenic factors were downregulated by probiotics administration (117). An ongoing clinical trial, NCT05032014, is investigating the potential of probiotics to enhance the efficacy of PD-1 inhibitors in liver cancer patients (95, 100).

While the therapeutic potential of probiotics is compelling, further studies are needed to explore their specific mechanisms and effectiveness in human HCC cases. Advancements in microbiome research and precision medicine may pave the way for integrating probiotics as cost-effective and safe adjunctive therapies in HCC prevention and treatment.

## 8 Conclusion

In summary, the gut microbiome plays a significant and multifaceted role in influencing the effectiveness of HCC treatments, through complex bidirectional interactions along the gut-liver axis. These interactions affect inflammation, the tumor microenvironment, metabolic pathways, and immune responses. Evidence from both animal models and emerging human studies demonstrates that the gut microbiome can modulate tumor responses to immunotherapy, and early data in HCC suggest that microbial signatures may serve as predictive markers for treatment outcomes and toxicities. Beneficial taxa such as *Akkermansia muciniphila* and higher microbial diversity have been associated with favorable immunotherapy responses, whereas dysbiosis may negatively impact drug metabolism, promote inflammation, and contribute to an immunosuppressive tumor microenvironment. Integrating microbial signatures with metabolomic data, immune markers, and clinical parameters may support more accurate patient stratification and guide personalized treatment decisions in HCC immunotherapy.

Despite this promise, the current evidence base remains limited. Most studies are underpowered, observational, or not specific for HCC, and few have established causal relationships or rigorously evaluated targeted microbiome interventions. Although strategies such as prebiotics, probiotics, dietary interventions, and FMT are being explored, their effects in HCC are not yet fully understood. Importantly, microbiome modulation carries potential risks, including unintended dysbiosis, altered drug metabolism, hepatic inflammation, and unpredictable microbial engraftment. These concerns are especially relevant for patients with cirrhosis or immunosuppression. Probiotics or FMT may pose additional safety concerns such as infection or bacteremia (118–120). Furthermore, interactions between microbiota-modulating therapies and existing HCC treatments require careful evaluation to avoid diminishing efficacy or exacerbating toxicity.

Effective integration of gut microbiota immunomodulation protocols into clinical practice will require personalized baseline microbiome profiling to identify patients most likely to benefit, standardized safety protocols, multidisciplinary collaboration among oncologists, hepatologists, microbiologists, and dietitians. Future studies should aim to identify specific microbial signatures associated with treatment response and toxicities, develop standardized workflows for microbiome sequencing, analysis and interpretation in HCC, characterize longitudinal microbiome dynamics following the administration of immunotherapies, and conduct large, prospective clinical trials to validate the role of gut microbiome in HCC treatment response. Understanding how specific microbial taxa and metabolites causally influence tumor immunobiology will be essential for designing rational and personalized microbiome-targeted interventions.

With continued research and clinical validation, gut microbiome modulation holds significant promise as an integral component of precision medicine in HCC. Harnessing the gut-liver axis to enhance immunotherapy efficacy may ultimately improve immunotherapy outcomes and patient prognosis.
